# Stacked ensemble deep learning for pancreas cancer classification using extreme gradient boosting

**DOI:** 10.3389/frai.2023.1232640

**Published:** 2023-10-09

**Authors:** Wilson Bakasa, Serestina Viriri

**Affiliations:** School of Mathematics Statistics & Computer Science, College of Agriculture, Engineering and Science, University of KwaZulu-Natal, Durban, South Africa

**Keywords:** stacking ensemble, deep learning, XGBoost, hyperparameters, classification, pancreas segmentation

## Abstract

Ensemble learning aims to improve prediction performance by combining several models or forecasts. However, how much and which ensemble learning techniques are useful in deep learning-based pipelines for pancreas computed tomography (CT) image classification is a challenge. Ensemble approaches are the most advanced solution to many machine learning problems. These techniques entail training multiple models and combining their predictions to improve the predictive performance of a single model. This article introduces the idea of Stacked Ensemble Deep Learning (SEDL), a pipeline for classifying pancreas CT medical images. The weak learners are Inception V3, VGG16, and ResNet34, and we employed a stacking ensemble. By combining the first-level predictions, an input train set for XGBoost, the ensemble model at the second level of prediction, is created. Extreme Gradient Boosting (XGBoost), employed as a strong learner, will make the final classification. Our findings showed that SEDL performed better, with a 98.8% ensemble accuracy, after some adjustments to the hyperparameters. The Cancer Imaging Archive (TCIA) public access dataset consists of 80 pancreas CT scans with a resolution of 512 * 512 pixels, from 53 male and 27 female subjects. A sample of two hundred and twenty-two images was used for training and testing data. We concluded that implementing the SEDL technique is an effective way to strengthen the robustness and increase the performance of the pipeline for classifying pancreas CT medical images. Interestingly, grouping like-minded or talented learners does not make a difference.

## 1. Introduction

Recent years have seen a remarkable expansion in automated medical image analysis (Wang, 2016; Litjens et al., 2017). Deep neural networks are one of the most well-liked and commonly used algorithms for computer vision problems (Goceri and Goceri, 2017; Fourcade and Khonsari, 2019; Wang et al., 2021). Deep convolutional neural network architectures serve as the foundation for this trend. These designs performed well with clinicians and demonstrated strong prediction ability (Currie et al., 2019; Lundervold and Lundervold, 2019; Zhou et al., 2021). Deep learning-based automated medical image analysis is currently a prominent academic topic being integrated into clinical workflow.

Medical image classification categorizes an entire image into specified classes according to a pancreatic cancer diagnosis or condition. To increase the accuracy of diagnoses or automate time-consuming procedures, these models are intended to be used as clinical decision support for physicians (Lee et al., 2017; Puttagunta and Ravi, 2021). Ensemble learning combines multiple machine learning models to produce superior results. The fundamental idea is a deep learning model combination, such as VGG16 (Kaur and Gandhi, 2019), Inception V3 (Alsabahi et al., 2018; Godasu et al., 2020), and ResNet34 (Dai et al., 2021; Xie et al., 2021), can produce more accurate results than any single machine learning model.

The world has moved toward a data-driven medical time, and artificial intelligence and machine learning are using this data to analyse diseases, forecast treatment results, and guide decision-making and drug development. The supervised machine learning classification approach is among the most popular and frequently used fields (Rauschert et al., 2020). It aids in sorting data into several categories. It has many uses, including speech recognition, image classification, fraud detection, and spam identification in email and other diagnostic tests and medical procedures (Dargan et al., 2020). Identifying a set of target classes (feature to identify in images) is the first step in the supervised learning process for classifying images. A model is trained to identify the target classes using labeled sample images. Rough pixel data was used as the model's input in early computer vision algorithms.

Medical image classification is a work in medical image analysis that entails categorizing medical images, such as X-rays, MRI scans, and CT scans, into different groups depending on the type of image or the presence of particular structures or disorders (Siddiq, 2020). One of the most significant issues in the field of image recognition is medical image classification, which aims to categorize medical images to aid physicians in the diagnosis of disease or further study. In general, there are two parts to medical image classification. The first step is to take the image and extract its useful elements. The second phase entails using the features to create classification models for the dataset of images. A tough, tedious, and time-consuming operation, classifying medical images into multiple classes traditionally required clinicians to apply their professional skills to extract features (Goel and Adak, 2021). This method is likely to produce unstable or unpredictable results. The application research for medical image categorization has had significant worth compared to previous studies.

Compared to individual models, ensemble approaches have higher predictive accuracy (Sundaramurthy et al., 2020). The approaches are particularly helpful when a dataset contains linear and non-linear data types since they combine several models to manage this data. Through ensemble approaches, bias and variance can be decreased, and the model is typically neither under nor overfitted (Zhang et al., 2022). Always less noisy and more stable is an ensemble of models. Utilizing an ensemble of models rather than a single model was the main objective while developing our proposed SEDL model to boost robustness and performance. The spread or dispersion of the forecasts and model performance decreases, while an ensemble can produce superior predictions and performance than any contributing model.

Models are also known as learners. Each learner is regarded as “weak” on their own. On the other hand, a robust model can be created when several weak learners are linked in some way. In our case, this robust model—SEDL—will be called an ensemble model. The models should be as diverse as possible. Each model may perform well on different subsets of the data. When the individual models are combined, their flaws are balanced out. The premise of ensemble models is that there is greater strength in multiple models than in a single one. In the approach section, we outline our suggested SEDL model and go into ensemble learning techniques (Ganaie et al., 2021). The experiments section describes our experiments and the procedures we used to develop and evaluate the model. The results section reviews the findings and some of the performance metrics we employed. Lastly, the concluding section provides a succinct summary.

## 2. Related work

Recent research has shown that ensemble learning algorithms are a significant component of the most effective and precise medical images categorization pipelines (Pintelas and Livieris, 2020; Xue et al., 2020; Yang et al., 2021a; Müller et al., 2022). Finding a model that maximizes prediction accuracy is the goal of machine learning. The method to combine numerous models into a better predictor closer to an optimal model was developed because it is challenging to determine the optimal model. The combining of models to provide improved prediction performance is what is referred to as ensemble learning. Deep ensemble learning (Pratiwi et al., 2021; Müller et al., 2022) incorporates ensemble learning techniques into a deep learning pipeline. Several recent research studies have effectively applied this method to increase a pipeline's performance and resilience for classifying medical images.

In the study (Barstugan et al., 2020), coronaviruses are categorized into two stages. Before this, the classification algorithm was applied to four subsets without first extracting the feature. Before SVM classification, the subsets underwent a vectorization step. The second stage involved the extraction of features using five distinct feature extraction methods, including the Discrete Wavelet Transform (DWT) (13), the Gray Level Size Zone Matrix (GLSZM) (12), Local Directional Patterns (LDP) (10), the Gray Level Run Length Matrix (GLRLM) (11), and the Gray Level Cooccurrence Matrix (GLCM) (7-9). SVM was used to classify the characteristics after that (14). The classification phase included cross-validation methods of 2x, 5x, and 10x. The mean classification results were found once the cross-validations were finished. The best classification accuracy was obtained as 99.68% with 10-fold cross-validation and GLSZM feature extraction method.

A convolutional Support Vector Machine is suggested in Özkaya et al. (2020b) and can categorize computed tomography images automatically. The CSVM model is trained from scratch instead of the pre-trained Convolutional Neural Networks trained with the transfer learning method. The dataset is separated into two parts: training (75%) and testing (25%) to assess the effectiveness of the CSVM approach. Three distinct numbers of SVM kernels are contained in each block of the CSVM model. With 94.03% ACC, 96.09% SEN, 92.01% SPE, 92.19% PRE, 94.10% F1-Score, 88.15% MCC, and 88.07% Kappa metric values, the performance of pre-trained CNN networks and CSVM models are compared. The CSVM (77, 33, 11) model performs the best.

According to empirical data, ensemble learning-based pipelines are often preferable because they combine the capabilities of various models to concentrate on distinct aspects while compensating for each model's specific limitations (An et al., 2020). It is still unclear whether ensemble learning models are useful in deep learning-based medical image categorization pipelines. Although generic ensemble learning is not new, the literature has yet to investigate how ensemble learning strategies affect deep learning-based classification. Few publications have begun to examine the deep ensemble learning field, unlike the authors who provide thorough reviews on general ensemble learning, such as according to Ganaie et al. (2021). While Sagi and Rokach (2018) and Kandel et al. (2021), they provide descriptions or analyses of general deep ensemble learning methods, while Cao et al. (2020) investigated deep learning-based ensemble learning methods in bioinformatics. In this study, we aim to create a SEDL pipeline to demonstrate the effect of ensemble learning techniques on deep convolution neural network performance for medical image categorization. We wish to evaluate the performance of deep learning techniques as base learners for XGBoost (Ramaneswaran et al., 2021) to uncover potential performance gains.

Firstly, the authors, Öztürk et al. (2021), use typical image augmentation on minority classes, which involves rotating, scaling, and other image modifications. The total number of photographs is now 260 more than before image enhancement. Fourth, four manually produced features are selected from all of the images. Seventy-eight features are created for each image by combining these feature vectors. The 78 feature vectors from the 260 images are then oversampled using the SMOTE technique. This methodology results in 495 feature vectors. Using these feature vectors, sAE and PCA are both trained. Out of a total of 78 features, 20 features were selected in this study using the sAE and PCA approaches. To classify data, SVM is trained using 495 vectors with 20 characteristics. The depth of the unbalanced structure in the dataset leads to the requirement for image augmentation and data over-sampling. Only two images will be created in many classes if image augmentation is the only technique used, and these two images will be nearly identical. Overfitting within the class takes place here. When solely employing a synthetic data oversampling method, performance data is obtained that is only a simulation of real-world performance, which may not improve. Two data replication methods are therefore combined for this purpose.

For detecting COVID-19 (Özkaya et al., 2020a), where early diagnosis is crucial for human life, CT image features are retrieved using the convolutional neural network architecture, currently the most successful image processing technique. Representational power is improved by fusing data and four CNN architectures' output features. Finally, the features integrated with the feature ranking algorithm are sorted, and their length is decreased. The dimensional curse is averted in this way. A subset was produced from 16 16 (Subset-1) and 32 32 patches taken, combining the predictions sent into the XGBoost (Liew et al., 2021) as a new train set; the ensemble prediction is the approach for use during the training and testing phases. The data that has been processed was then categorized using the Support Vector Machine technique.

## 3. Methods and techniques

Deep ensemble learning is commonly defined as assembling a group of many predictions derived from various deep convolutional neural network models (Kandel et al., 2021). Ensemble learning must now be defined in deep learning as combining data, most frequently predictions, for a single inference due to recent breakthrough methodologies. This data or these forecasts may come from a single model, several independent models, or none. Using numerous unique models in a stacking method, we investigated the performance impact of ensemble learning techniques in this investigation. The ensemble prediction is obtained by combining the predictions sent into the XGBoost (Liew et al., 2021) as a new train set.

On the TCIA dataset, the SEDL classification architectures we employed in this study had good results. VGG16, ResNet34, and Inception V3 are the architectures selected for first-level predictions. At the second level prediction, we used XGBoost. These networks have been specially designed for classifying pdac CT images and have undergone extensive training. Each of these networks has a different hyper-parameter value that is optimized. Here's a basic description of these architectures:

### 3.1. Deep ensemble learning

In a machine learning paradigm known as ensemble learning, multiple learners are trained to solve the same problem (Sagi and Rokach, 2018; Ganaie et al., 2021). In contrast to traditional deep learning approaches, which attempt to learn one hypothesis from training data, ensemble methods attempt to generate and combine several hypotheses. Several students who make up an ensemble are typically referred to as base students. An ensemble's capacity for generalization is typically substantially greater than that of basic learners. Deep ensemble learning (An et al., 2020; Pintelas and Livieris, 2020) is intriguing because it can transform weak predictors that are only marginally better than random guesses into strong predictors that can make extremely precise predictions. In this sense, “base learners” and “weak learners” are synonyms. To create heterogeneous learners, we used a variety of learning algorithms. Since there is no one base learning algorithm in heterogeneous learning, some people prefer the terms “base learners” over “individual learners” or “component learners.”

By considering the nature of machine learning as exploring a hypothesis space for the best accurate hypothesis, Gu et al. (2019), Alam et al. (2020), and Wu et al. (2020) provided explanations for why the generalization ability of an ensemble is typically significantly stronger than that of a single learner. The training data may not contain enough details to identify only one best learner, which is the first justification. For instance, multiple students might perform equally well on the practice data set. Therefore, it could be preferable to combine these learners. The second concern is that the learning algorithms' search methods may be flawed. For instance, even if there is a single best hypothesis, it could be challenging since applying the algorithms yields less-than-ideal hypotheses.

### 3.2. Stacking

**Figure 1:**
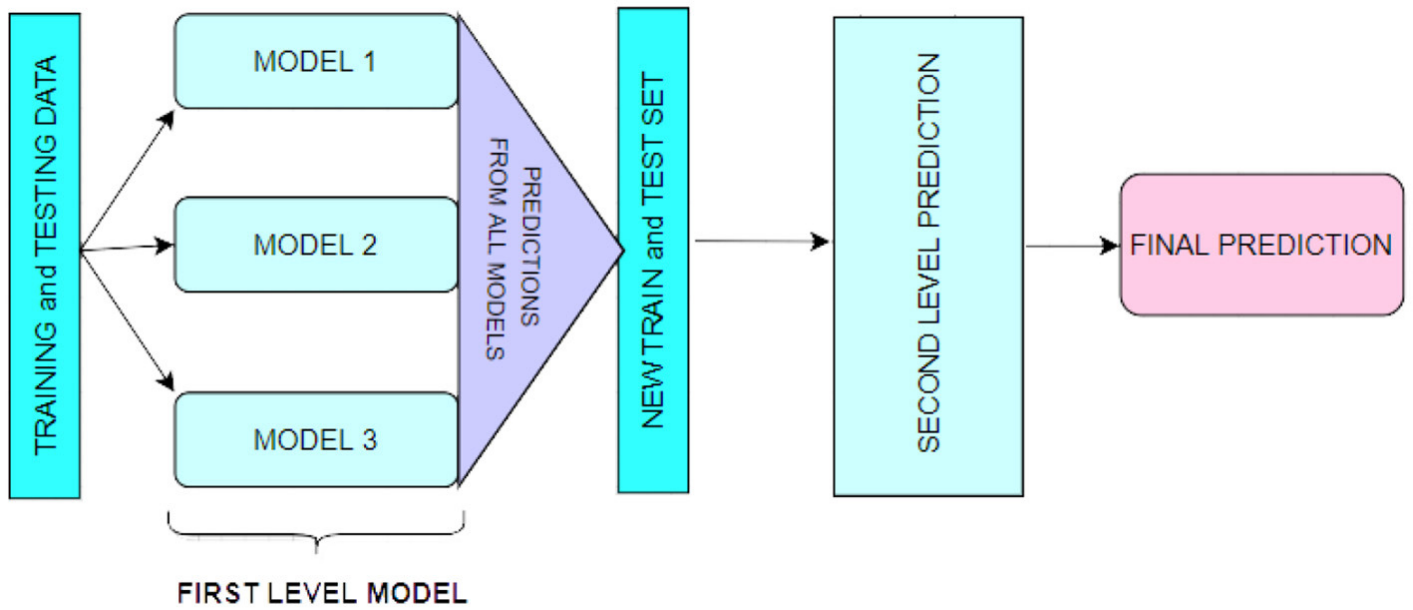
The demonstration of a stack for first-level prediction.

A technique for combining multiple regression or classification models, as illustrated in Figure 1. Bagging and boosting are the two most well-known ensemble modeling techniques. Bagging (Chatterjee et al., 2019; Liu and Long, 2019) allows for averaging several comparable models with high volatility to reduce variance. Increasing (Chatterjee et al., 2019; Iranzad et al., 2022) generates many incremental models to reduce bias while minimizing variation.

Stacking is a different paradigm. Stacking's goal is to explore a space of several models for the same problem. A learning problem can be approached using several models to learn a portion of the problem but not the entire problem space. As a result, we can generate several learners and use them to generate intermediate predictions, one for each learned model. Then we add a new model that targets the same target as the intermediate forecasts.

The name of the last model comes from the claim that it is layered on top of the others. As a result, we may enhance our total performance, and frequently we produce a model superior to each intermediate model.

### 3.3. Max voting implementation

Voting is effective because it gives most models' opinions more weight than the vote on a single model. When the models may vote on discrete possibilities, maximum voting is utilized. The choice is the proposal that has received the most votes. It is employed to solve classification issues. The option with the most votes is chosen. Each deep learning model casts a vote. Max voting comes in two flavors: harsh and soft. We attempt to predict five classes, T0 (no cancer signs), T1 (tumor ≤ 2 cm), T2 (tumor > 2 cm ≤ 4 cm), T3 (tumor > 4 cm), and T4 (tumor spread), using three basic classifiers, VGG16 (Jiang et al., 2021), ResNet34 (Godasu et al., 2020), and Inception V3 (Xie et al., 2021). After training the base classifier models, we attempt to predict a single point's class.

### 3.4. Models used in ensemble

The following models were used in the first level prediction stage to implement the ensemble:

#### 3.4.1. VGG16

Oxford University developed one of the most popular deep learning architectures, VGG16 (Kaur and Gandhi, 2019). It has 41 strata that have been disturbed: 13 convolutional layers (Conv.), 16 weight layers, and 3 Fully Connected layers. All Conv Layers in VGG16 use a tiny 3 × 3 kernel (filter) with stride 1. Conv. Max pooling layers always follow layers. Three fixed-size channels of 224 × 224 pixels make up the input for VGG16.

The three Fully Connected layers in VGG16 (Chhabra and Kumar, 2022) have various depths. The soft-max layer controls the probability specified for the input image and serves as the output layer. If the weights are started randomly, VGG16 (Jiang et al., 2021) requires extensive training, just like any pre-trained model. Consequently, transfer learning methods are generally used in Convolutional Neural Network (CCN) models. TL describes a technique where a model developed for one activity is used in some way for another comparable task.

#### 3.4.2. ResNet34

Given that neural networks are models of the human brain and how it thinks, it stands to reason that deeper networks would be used to mimic the deeper thinking required to solve difficult problems. The issue of vanishing gradients is the fundamental issue facing deep networks (Godasu et al., 2020).

ResNet34 (Alsabahi et al., 2018) is a neural network that solves the issue of training deep learning networks using skip connections. It “skips” several convolutional layers in each basic network block, providing alternate paths for the original and derived data and enabling training to be completed more quickly. The following equation, which describes such skip connections, adds the outputs of the preceding blocks to the ones that follow:


(1) $$\begin{array}{l} y=F\left(x\right)+x \end{array}$$

When F is the residual function, as in Equation (1), *x* is the input, and *y* is the output. Two convolution layers, a pooling layer (3 × 3 size), a (ReLU) activation function, and batch normalization comprise each basic block. ResNet has significantly improved neural network performance by stacking more layers onto neural networks to provide a deeper architecture and, thus, deeper learning instead of shallower learning. The ResNet-34 (Talo et al., 2019; Nayak et al., 2021) (ResNet with 34 layers) comprises a fully connected layer, a max-pooling layer (3 × 3 size), a layer with average pooling, and 33 convolutional layers.

#### 3.4.3. Inception V3

In 2014, Google proposed the GooLeNet network, which is a CNN. It employs the Inception network topology, which reduces the number of network parameters while increasing network depth. As a result, it is frequently used in image classification jobs. Because its primary component is the (Li et al., 2019; Singh et al., 2020), the GoogLeNet network is also known as the Inception network. The four primary versions of Google Net are Inception v1 (2014), Inception v2 (2015), Inception v3 (2015), Inception v4 (2016), and Inception-ResNet (2016). The Inception module typically includes one maximum pooling and three varying-sized convolutions.

After the convolution operation, the channel is aggregated for the net output of the preceding layer, and the non-linear fusion is then carried out. This method can avoid over-fitting while enhancing the network's expression and flexibility to various scales. The main component of Inception v3 is a network structure created by Keras that has already been trained on Image Net. Three channels and a preset image input size of 299*299 are used. Unlike the Inception v1 and v2 network structures, the Inception v3 network structure splits huge volume integrals into smaller convolutions using a convolution kernel splitting technique (Ganaie et al., 2021).

### 3.5. XGBoost

The sophisticated use of the gradient boosting method is called XGBoost. An extremely successful deep learning algorithm is XGBoost (Ramaneswaran et al., 2021). Compared to other gradient-boosting approaches, XGBoost is nearly ten times faster and has strong predictive power. Additionally, it contains a range of regularizations that lessens overfitting and enhances general performance. It is often referred to as the “regularized boosting” technique. Due to the following characteristics, XGBoost (Liew et al., 2021) is compared to other approaches as being superior:

1. Regularization (Shilong and Diaru, 2021):

- Unlike XGBoost, the standard Gradient Boosting Machine (GBM) implementation does not regularize.
- As a result, XGBoost also aids in lowering over-fitting.

2. Parallel Processing (Henriques et al., 2020):

- In comparison to GBM, XGBoost uses parallel processing and is faster.
- XGBoost also supports Hadoop implementation.

3. High Flexibility (Zhang D. et al., 2021):

- With the help of XGBoost, users can create unique optimization goals and assessment standards, giving the model an entirely new dimension.

4. Handling Missing Values (Zhang et al., 2020):

- A built-in procedure in XGBoost can deal with missing values.

5. Tree Pruning (Sagi and Rokach, 2021):

- After splitting the tree to the maximum depth chosen, XGBoost begins to prune the tree backwards and removes the splits above without benefit.

6. Built-in Cross-Validation (Wang et al., 2020):

- Because the user can perform cross-validation at each stage of the boosting process, obtaining the precise ideal number of boosting iterations in a single run is simple with XGBoost.

### 3.6. Proposed model

Stacking (Wang et al., 2019) is a sophisticated ensemble learning model. The fundamental idea behind stacking is that we base our future forecasts on the base data's derivative models. Now, the outcomes would also be comparable if the models were. To better understand the outcome, we purposefully chose various models, as those models may have learned some aspects of the data more effectively. Stacking (Haq et al., 2022) takes diverse weak learners into account. Stacking combines several weak learners by training a meta-model to produce predictions based on the numerous predictions that these weak learners returned.

**Figure 2:**
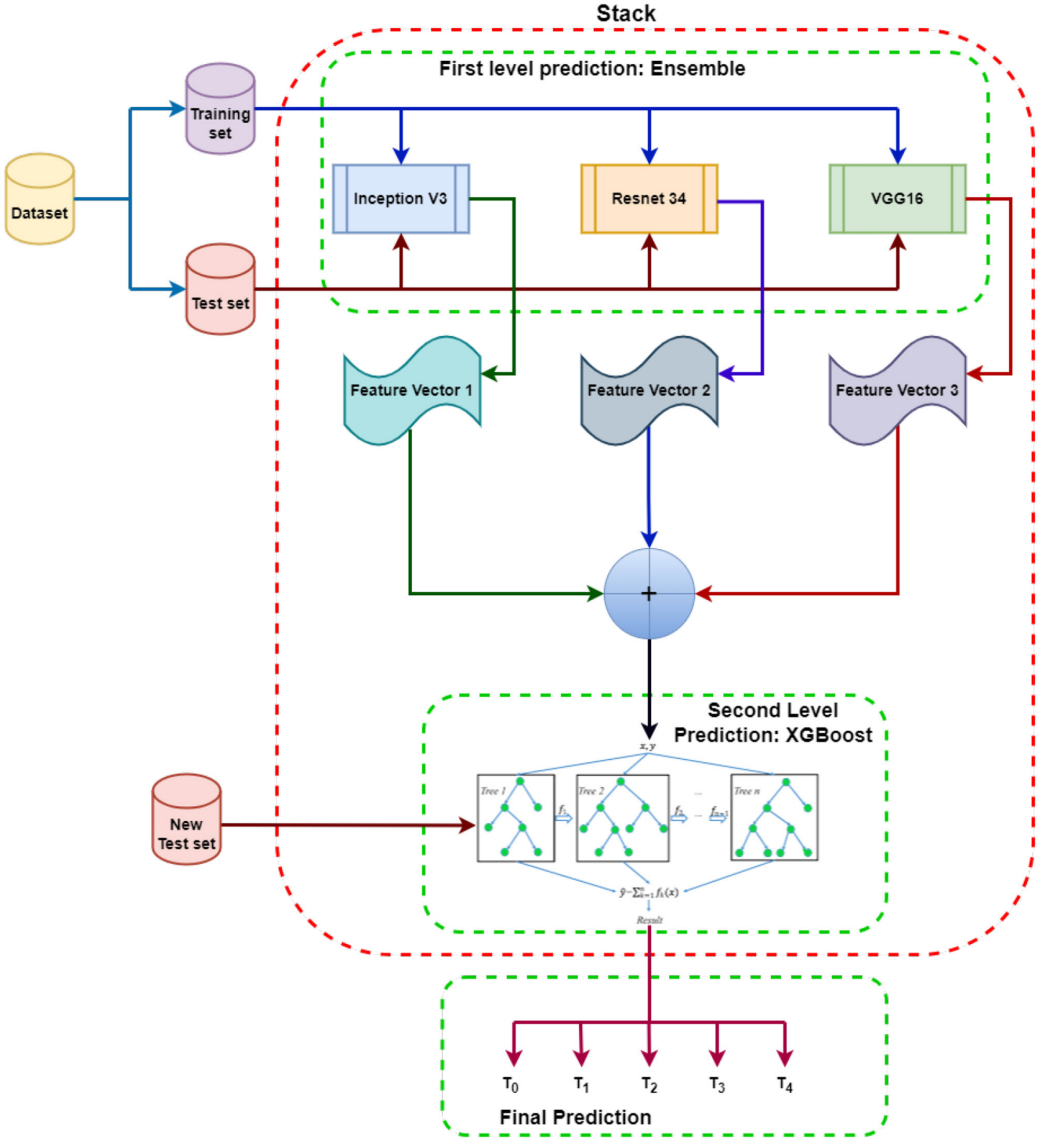
Proposed SEDL Model: Each model at the first level prediction produces its own independent set of the base feature vector. These features from each ensemble model are then fused to make the meta feature vector that will be input into XGBoost to learn from and evaluated using the new test set. The results are different classes depending on the prediction based on the features obtained.

The suggested SEDL framework has two layers, depicted in Figure 2. Each basic classifier in the first layer—VGG16, Inception V3, and ResNet34—was trained using training data. Different classifiers in the representation learning technique of stacking express heterogeneity for various features (Tang et al., 2019). High diversity and high accuracy are two conditions the first layer's basic classifiers must meet to learn features from the raw input effectively. The three base classifiers are highly good at solving the non-linear problem, but their modeling approaches are very dissimilar.

Inception V3, VGG16, and ResNet34 are used to make first-level predictions from the dataset, divided into training and test sets. The three models are each trained from scratch on the dataset to generate feature predictions for pdac CT images. The feature predictions from the first-level prediction models are combined and sent as input to the XGBoost-based second-level prediction. The new test data is used to evaluate the performance and progress of the algorithm training and adjust or optimize it for improved results. XGBoost will make the final prediction and classifications.

The training grounds for ensemble classification systems are developing various single classification methods to solve the same job and the subsequent aggregation of their results using a particular combiner. The suggested model uses transfer learning for image classification (Jaiswal et al., 2021). It holds that a model can effectively function as a generic model of the visual world if trained on a sizable and general enough dataset. By training a big model on a big dataset, we can use these learnt feature maps instead of starting from scratch. Take advantage of a prior network's representations learned to identify significant aspects in fresh data. On top of the pre-trained model, add a fresh classifier trained from scratch to reuse the feature maps previously learned for the dataset. Following that, the classifier receives the features learned from the ensemble models. Transfer learning has made it possible to train deep neural networks even with little or no data by utilizing the capacity to reuse previous models and their understanding of new issues (Iman et al., 2023). Transfer learning has the advantages of using fewer data, requiring the classifier to train more quickly, and performing better on neural networks.

We ultimately integrated all three models to produce the first layer of the stacking model due to their similarities and differences, as well as the positive results from cross-validation. Strong generalization skills are necessary for the meta-learner in the second layer to rectify the bias of various learning algorithms toward the training set and prevent the over-fitting effect through aggregation. Therefore, we used XGBoost (Shilong and Diaru, 2021) to benefit from its second-level meta-learner generalization capacity. This approach employs the greatest likelihood method to estimate the parameters after assuming all data follow the logical distribution.

A broad range of hyperparameters offered by the XGBoost algorithm needed to be adjusted for a better classification model. A machine learning model's overall performance and behavior can be enhanced by hyperparameter adjustment (Zhang B. et al., 2021). It is a particular parameter chosen before learning that occurs outside the model. If the loss function is not minimized, improper results may follow from a lack of hyperparameter tweaking. We aim to have as few errors as feasible produced by our model. Finding a collection of ideal hyperparameter values that maximizes the model's performance, minimizes loss, and generates superior outputs is the main goal of hyperparameter tweaking. We had to adjust the XGBoost hyperparameters like model_colsample_bytree, model_gamma, model_learning_rate, model_max_depth, model_min_child_weight, model_n_estimators, model_reg_lambda, and model_subsample, to achieve better classification results.

## 4. Experiments

We used logic to create parallel ensemble models (Wei et al., 2020) that take advantage of the basic learners' independence (rather than the Sequential ensemble models, which employ logic to leverage the dependence between the base learners.). As a result, labeling errors generated by one model are distinct from those made by a different independent model. The ensemble model can then average out the mistakes as a result. Table 1 shows some of the computational specifications required for the experiments.

**Table 1:** Hardware and software specifications for the experiments.

**Hardware**	**Software**
Graphical Processing Unit (GPU)	Programming language: Python version 3.9
RAM: 32 gigabytes	Backend: Tensorflow GPU
Processor: core i5 2.2 gigahertz	Deep learning API: Keras GPU
NDVIDIA 16 gigabytes RAM	
Hard drive: 500 gigabytes	

**Figure 3:**
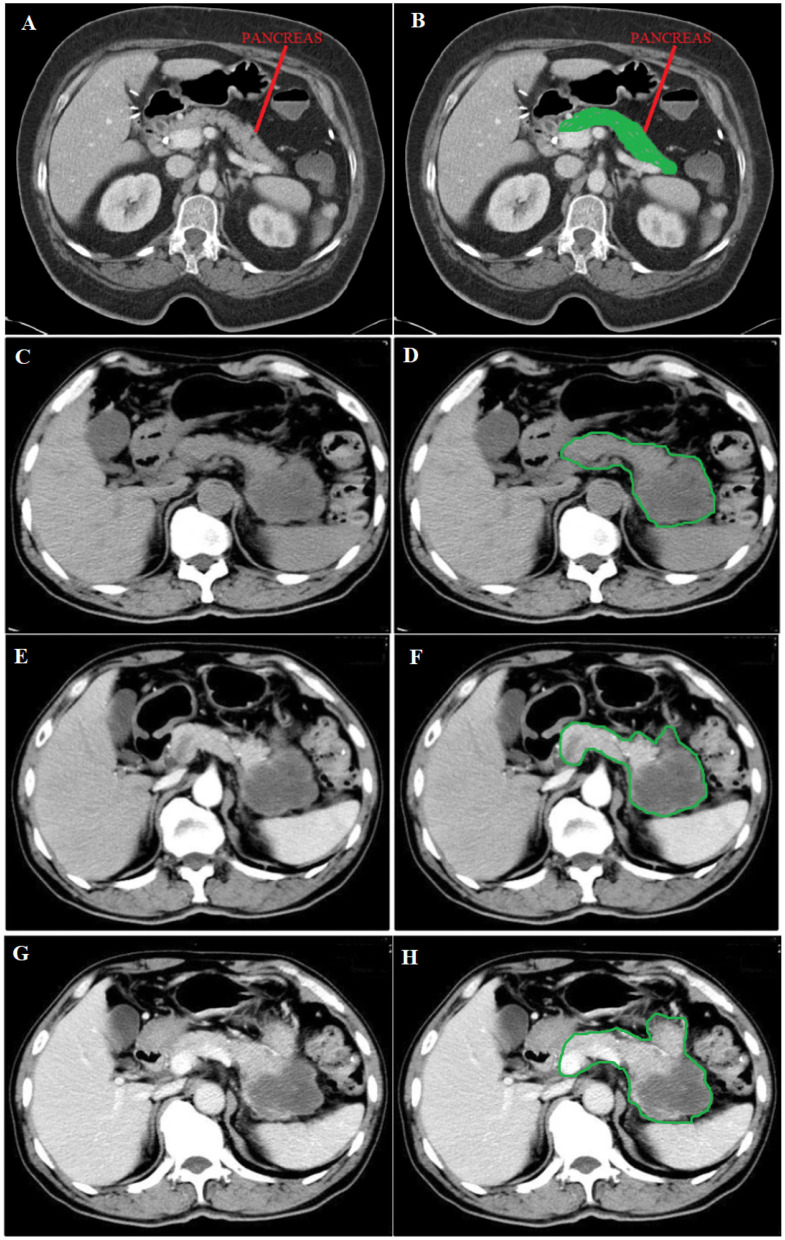
**(A)** Normal image of the pancreas, **(C)** Unenhanced phase, **(E)** Arterial phase, and **(G)** Portal phase. **(B)** in green shows the position of the normal pancreas, while **(D, F, H)** in green circulation show the spread of pdac. Source of images: Roth et al. (2016).

The data for classifying pdac contains CT scans from 80 subjects, and 222 images were used. Two hundred twenty-two images were used, of which 0.80 was train data, and 0.20 was test data. As shown in Figure 3, there were different stages of pdac:

- *T*0 = *Image*_*A*_*stage*,
- *T*1 = *Image*_*C*_*stage*,
- *T*2 = *Stage*_*between*_*Image*_*C*_*and*_*E*,
- *T*3 = *Image*_*E*_*stage*,
- *T*4 = *Image*_*G*_*stage*.

Image A shows a Normal pancreas, while Image C shows an unenhanced stage of pdac. Image E shows the arterial phase, a contrast-enhanced CT series, in which the contrast is still in the arteries and has not reached the organs and other soft tissues. Image G shows the portal phase, a contrast-enhanced CT series at its peak enhancement.

The steps followed to do the experiments on each base model are in the Algorithm 1, which shows how VGG16, ResNet34 and Inception V3 are trained and used in the first-level prediction classification phase.

**Algorithm 1:** Model algorithm: The model performs an ensemble classification using three models, that is, VGG16, ResNet34 and Inception V3. These steps are followed to train each model on the same dataset. A confusion matrix and accuracy score are produced for each model.

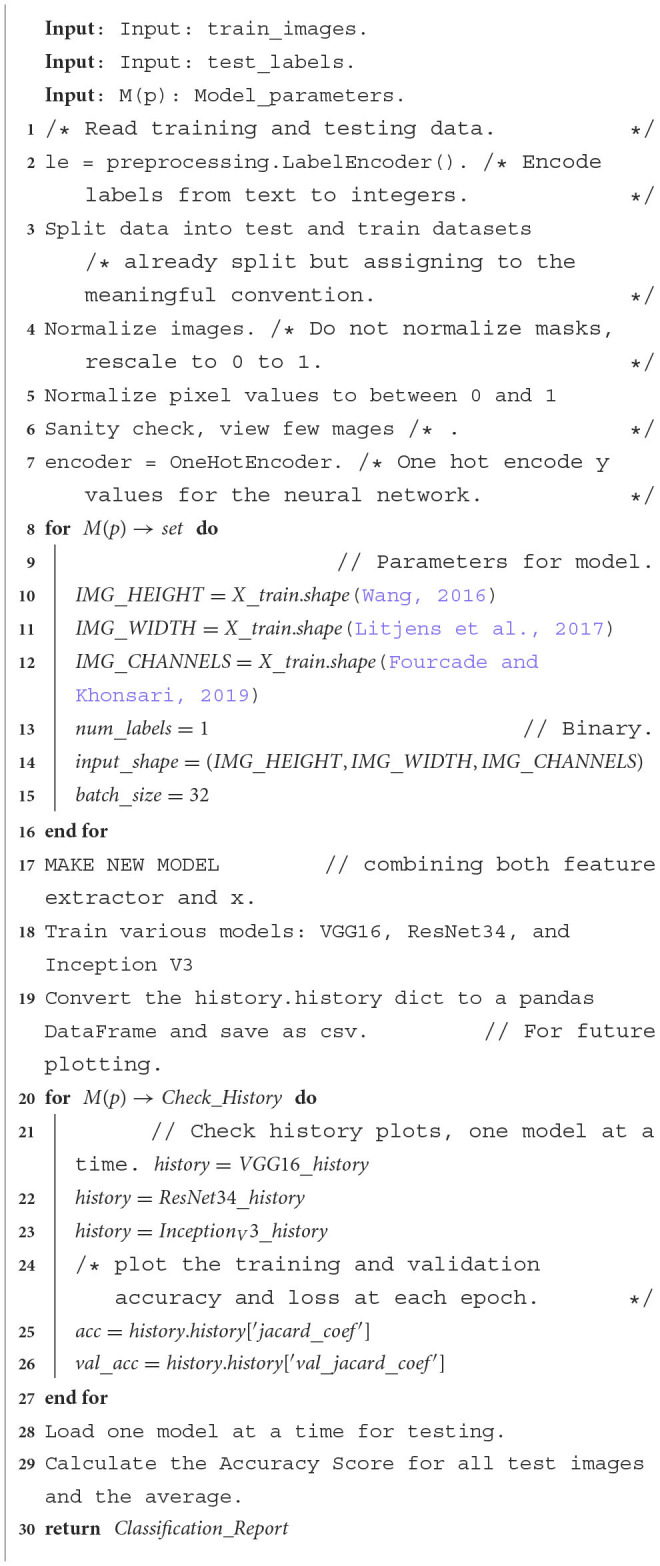

We then ensemble the predictions from base models to be the new train set for the final classifier in the second level prediction phase, tested for XGBoost, LGBM and Random Forest, as shown in Algorithm 2 steps.

**Algorithm 2:** XGBoost classifier: Steps followed to implement XGBoost, LGBM, or Random Forest as an image classifier at the second level prediction phase.

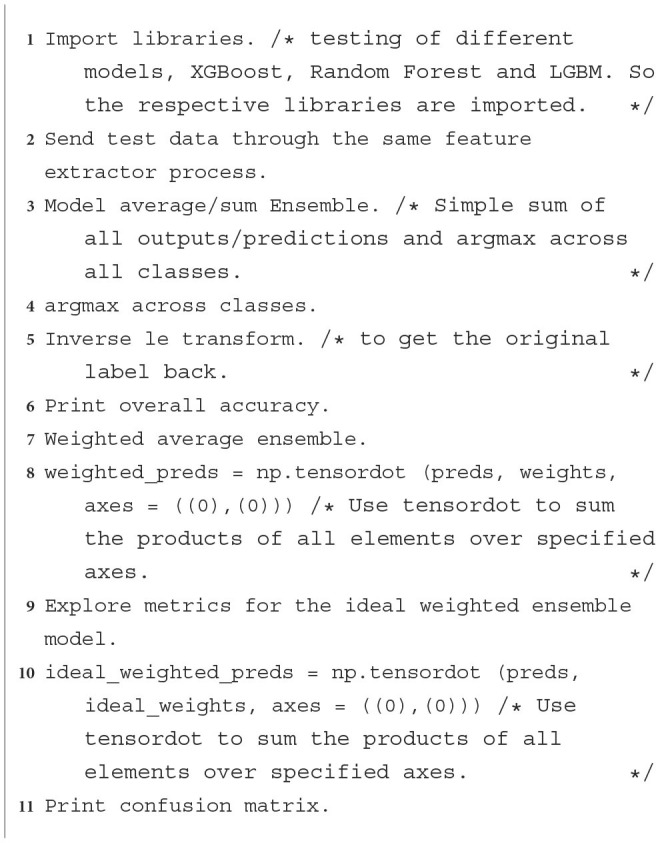

## 5. Results

Compared to using a single model, ensemble learning enhances model results by combining the knowledge and approach of multiple models. For ensemble models to function, several weak learners must be combined into one strong learner, which helps by lowering bias, reducing variation, or increasing prediction accuracy.

### 5.1. XGBoost hyperparameters tuning

A wide variety of hyperparameters are available with the XGBoost algorithm (Sommer et al., 2019). For the XGBoost model to work better and to its full potential, we need to be able to adjust these hyperparameters. Optimizing a machine learning model's hyperparameters is essential for enhancing its general behavior and performance. It's a parameter chosen before the learning process and takes place independently of the model. Table 2 shows the results after adjusting the parameters to have optimal results from the XGBoost classifier at second-level prediction after implementing the ensemble technique at first-level prediction. The best model learning rate was 0.085, with an accuracy of 0.9677. An accuracy of 0.9643 was achieved at a model n_estimator of 250. The model_colsample_bytree at 0.9 achieved better accuracy of 0.9686 than the default model_colsample_bytree of 1. The model subsample was best at 0.8 with an accuracy of 0.984. Model gamma at 4.5 was the best, with 0.989 accuracy.

**Table 2:** XGBoost parameter tuning at a second level prediction: XGBoost as the classifier had to be tested using different parameters to obtain optimal accuracy.

**Constant parameter**	**Changing parameter**	**Parameter value**	**Accuracy**	**Macro avg**	**Weighted avg**
*mcb* = 0.8, *mmd* = 5, *mmw* = 1, *ms* = 0.8	mlr	0.01 • 0.05 • 0.085 • 0.1 • 0.15	0.9345 • 0.9442 • 0.9677 • 0.9475 • 0.8876	0.9345 0.9542 0.9695 0.9434 0.8844	0.9298 0.9542 0.9676 0.9428 0.8830
	mne	800 • 550 • 250 • 100	0.9468 • 0.9547 • 0.9643 • 0.9475	0.9453 • 0.9567 • 0.9665 • 0.9444	0.9332 0.9579 0.9629 0.9430
*mlr* = 0.085, *mmd* = 5, *mmw* = 1, *mne* = 250	mcb	0.6 • 0.8 • 0.9 • 1	0.9553 • 0.9574 • 0.9686 • 0.9578	0.9555 • 0.9574 • 0.9678 • 0.9572	0.9552 0.9574 0.9678 0.9575
	ms	0.6 • 0.8 • 0.9 • 1	0.9276 • 0.9840 • 0.9733 • 0.9693	0.9243 • 0.9840 • 0.9641 • 0.9679	0.9231 0.9822 0.9624 0.9661
*mcb* = 0.9, *mlr* = 0.01, *mmd* = 5, *mmw* = 1, *mne* = 250, *ms* = 0.8	mg	0 • 1 • 3 • 4.5 • 5	0.9445 • 0.9442 • 0.9713 • 0.9895 • 0.9856	0.9445 • 0.9432 • 0.9671 • 0.9889 • 0.9836	0.9398 0.9428 0.9660 0.9880 0.9839

This table shows, mlr (model_learning_rate), mcb (model_colsample_bytree), mg (model_gamma), mmd (model_max_depth), mmw (model_min_child_weight), mne (model_n_estimators), ms (model_subsample), Accuracy, Macro Avg (Macro Average), and Weighted Avg (Weighted Average).

If the loss function is not minimized, findings without proper hyperparameter adjustment may be unreliable. We want as few errors as possible to be produced by our model. Hyperparameter tuning aims to find ideal hyperparameter values that maximize model performance, minimize loss, and create superior outputs (Putatunda and Rama, 2018).

### 5.2. Averaging and weighted average

When averaging, multiple forecasts are made for each data point, similar to the max voting method. In this approach, the final prediction is made by averaging the results of all the models. Probabilities can be computed for classification issues using averaging.

Weighted Average is an extension of the averaging method. Different weights are assigned to each model, indicating the significance of each model for prediction. For instance, the responses from these two models will be given greater weight than those from the other model if two of the models are critics and one has no prior expertise in this subject. Table 3 shows the averaging and weighted average for the three models tested as classifiers at the final stage. XGBoost had the best ensemble average and ensemble weighted average at 0.987 and 0.981, respectively. The averaging for the ensemble shows a better improvement of results than when using individual models at first-level prediction when implemented at the second-level prediction.

**Table 3:** Average score table showing the averaging of using a classifier at second level prediction after implementing individual models without ensemble at first level prediction.

**Classifier**	**Inception V3 averaging**	**VGG16 averaging**	**ResNet-34 averaging**	**Avg ensemble**	**W avg ensemble**
XGBoost	0.942	0.979	0.964	0.987	0.981
LGBM	0.905	0.953	0.948	0.955	0.951
RF	0.940	0.942	0.939	0.952	0.949

Avg Ensemble (Average Ensemble) and W Avg Ensemble (Weighted Average Ensemble) for different model classifiers at second level prediction are also shown after implementing ensemble at first leave prediction.

### 5.3. Confusion matrix

**Figure 4:**
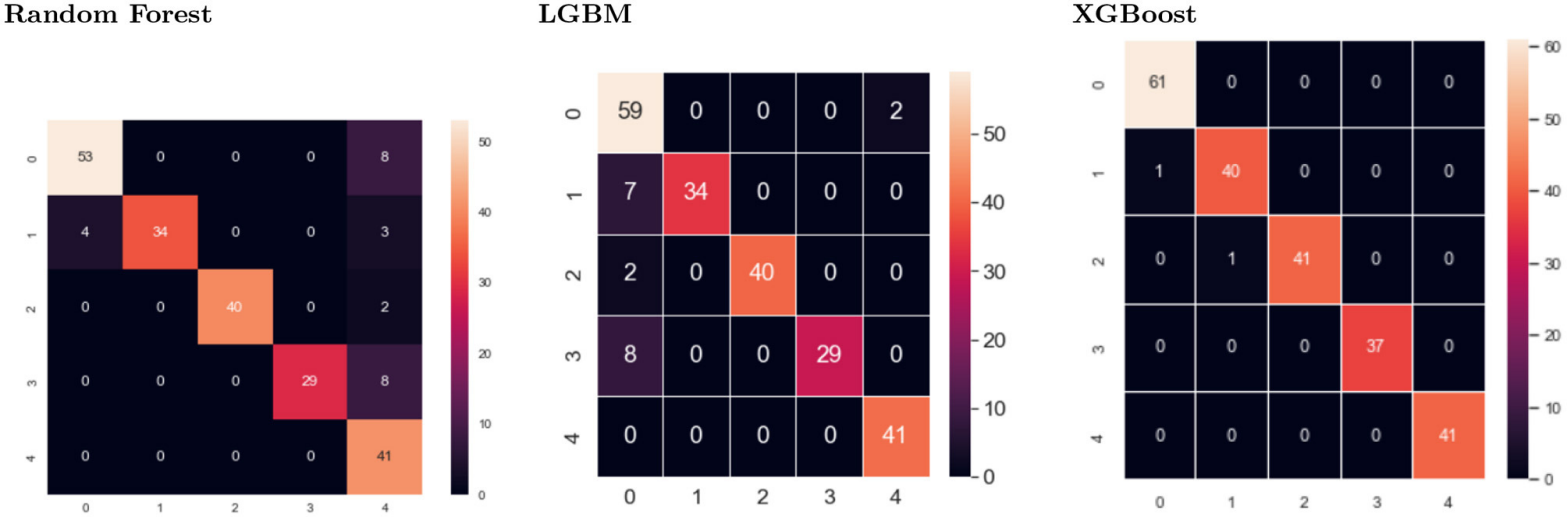
Confusion matrix for Random Forest, LGBM, and XGBoost as the classifiers.

The confusion matrix is made up of five target classes. A 5*x*5 matrix is used to evaluate how well a proposed SEDL classification model performs at second-level prediction, as shown in Figure 4 using different models. XGBoost showed less misclassification than the other models. The deep learning model's predictions are compared to the target values in the matrix, providing a comprehensive understanding of our classification model's effectiveness and the types of errors it makes.

The confusion matrix for XGBoost shows two negative images, while two hundred and twenty images are positive, out of two hundred and twenty images. Each column in the confusion matrix represents the instances of that projected class. Each row of the confusion matrix displays an instance of the real class. It provides insight into errors that are being made and errors made by a classifier.

### 5.4. Classification report

The primary classification metrics at second-level prediction are provided for each class in the classification report as in Table 4. The classification report (Luque et al., 2019) visualizer shows the model's precision, recall, F1 and support scores for the three models tested at second-level prediction after the ensemble is implemented at first-level prediction. In contrast to overall accuracy, which can hide functional deficiencies in one class of a multi-class problem, this provides a deeper insight into the classifier behavior.

**Table 4:** Classfication report for ensemble model showing Avg Ensemble (Average Ensemble), W Avg Ensemble (Weighted Average Ensemble), Accuracy, Macro avg (Macro average), and Weighted avg (Weighted average) for different models.

**Classifier**	**Class**	**Precision**	**Recall**	**F1 score**	**Support**
XGBoost	T0 • T1 • T2 • T3 • T4	0.98 • 1.00 • 1.00 • 1.00 • 0.95	0.98 • 0.98 • 0.98 • 1.00 • 1.00	0.98 • 0.99 • 0.99 • 1.00 • 0.98	61 41 42 37 41
	**Metrics**				
	**Metric**	**Precision**	**Recall**	**F1 score**	**Support**
	Accuracy • Macro avg • Weighted Avg	- • 0.99 • 0.99	- • 0.99 • 0.99	0.99 0.99 0.99	222 222 222
LGBM	T0 • T1 • T2 • T3 • T4	0.78 • 1.00 • 1.00 • 1.00 • 0.95	0.97 • 0.83 • 0.95 • 0.78 • 1.00	0.86 • 0.91 • 0.98 • 0.88 • 0.98	61 41 42 37 41
	**Metrics**				
	**Metric**	**Precision**	**Recall**	**F1 score**	**Support**
	Accuracy • Macro avg • Weighted Avg	- • 0.95 • 0.93	- • 0.91 • 0.91	0.91 • 0.92 • 0.92	222 222 222
RF	T0 • T1 • T2 • T3 • T4	0.71 • 1.00 • 1.00 • 1.00 • 1.00	1.00 • 0.59 • 0.95 • 0.84 • 1.00	0.83 • 0.74 • 0.98 • 0.91 • 1.00	61 41 42 37 41
	**Metrics**				
	**Metric**	**Precision**	**Recall**	**F1 score**	**Support**
	Accuracy • Macro avg • Weighted Avg	- • 0.94 • 0.92	- • 0.88 • 0.89	0.89 • 0.89 • 0.89	222 222 222

Precision can be thought of as a measure of a classifier's accuracy. Recall is a measure of a classifier's completeness; it is the classifier's ability to identify all positive instances correctly. The F1 score is a weighted harmonic mean of precision and recall, where 1.0 represents the best, and 0.0 represents the worst. The precision, recall, and f1-score are all greater than 0.98, implying that the SEDL model with XGBoost is a powerful classification tool. Support is the number of actual class occurrences in the specified dataset.

### 5.5. Training and validation loss

The training loss metric assesses how well a deep learning model fits the training data. In other words, it assesses the model's error on the training set. It should be noted that the training set is a subset of the dataset that was initially used to train the model. The training loss (Deepak and Ameer, 2019) is calculated by adding the sum of errors for each example in the training set. It is also important to note that the training loss is calculated after each batch, typically represented by plotting a training loss curve.

Validation loss (Dhillon et al., 2019), on the other hand, is a metric used to assess the performance of a deep learning model on the validation set. The validation set is a subset of the dataset set aside to test the model's performance. The validation loss is calculated similarly to the training loss by adding the errors for each example in the validation set. Furthermore, the validation loss is calculated after each epoch, indicating whether the model requires additional tuning or adjustments. For the validation loss, we created a learning curve.

**Figure 5:**
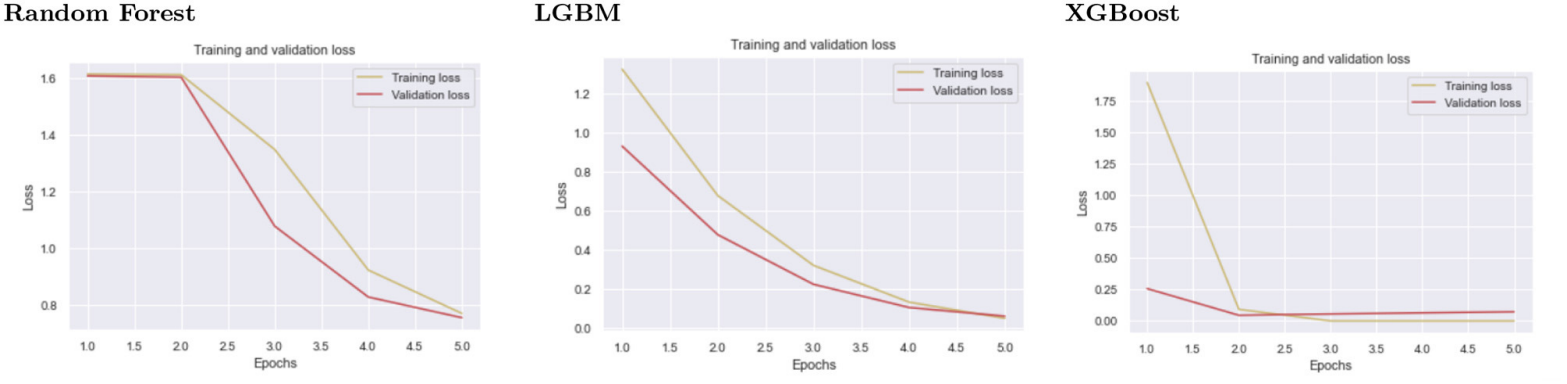
The graphs show the validation and testing loss graphs for the three models used as learners at the first level of prediction.

The second level classification by XGBoost displays good fit learning curves, which is the learning algorithm's goal, and exists between an overfit and underfit model. Figure 5 shows training and validation loss for different models at second-level prediction. A training and validation loss identifies a good fit that decreases stability with a minimal gap between the two final loss values, as shown by the XGBoost graph.

The model's loss is almost always lower on the training dataset than on the validation dataset, which implies that there will be some disparity between the train and validation loss learning curves known as the “generalization gap.” The plot of learning curves for XGBoost shows a good fit, indicated by:

- *T*he training loss plot decreases to the point of stability.
- *T*he validation loss plot approaches stability and has a small gap with the training loss.

### 5.6. ROC

**Figure 6:**
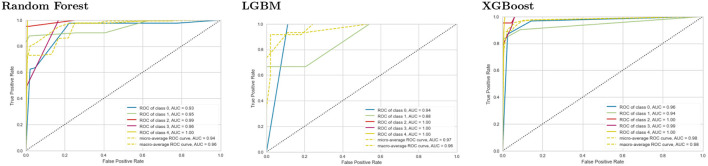
ROC curves for the Random Forest, LGBM, and XGBoost implementation at second level prediction. AUC is the Area under the ROC Curve.

A graph depicting the effectiveness of a classification model at all classification thresholds is called a ROC curve (receiver operating characteristic curve). Figure 6 shows the ROC curve for the three models tested at second-level prediction. The True Positive and False Positive rates are plotted on this curve. The chart demonstrates the connection between the true and false positive rates. It was decided to compare each class to every other using the One-vs-Rest methodology. XGBoost had an AUC (Area under the ROC Curve) of 0.98, which is best than the other models.

### 5.7. Discussion

The idea of stacked ensemble deep learning is used in our proposal of a unique deep learning framework for identifying pdac. AUC was 98.6%, F1 was 99.6%, and accuracy was 98.8% obtained from the studies. Using several neural network models trained on ImageNet (Chouhan et al., 2020), features from images are extracted in this method and fed into a classifier for prediction. They suggested an ensemble model incorporating all pre-trained models' outputs to achieve the best pneumonia recognition performance. This model surpassed individual models. On uncovered data from the Guangzhou Women and Children's Medical Centre dataset, their ensemble model achieved an accuracy of 96.4% and a recall of 99.62%. Our model SEDL performs end-to-end classification in two tiers by utilizing each model's ability to anticipate any hidden features in the pdac CT images.

According to Rajaraman and Antani (2020), a stacked ensemble of the top three retrained models shows promising performance (accuracy: 0.941; 95% Confidence Interval (CI): [0.899, 0.985], Area Under the Curve (AUC): 0.995; 95% CI: [0.945, 1.00]). The ensemble approaches' accuracy (*P* = 0.759) and AUC (*P* = 0.831) do not differ statistically, according to one-way ANOVA analysis. The classification of our SEDL model was enhanced by the knowledge conveyed through modality-specific learning of pertinent features. The SEDL model decreased the prediction variance and sensitivity to changes in training data. The outcomes of combining them outperform current technology.

Using chest x-ray and Canny edge detected images, Hijazi et al. (2019), demonstrate a deep ensemble learning for TB identification. This strategy increases the base classifiers' diversity of mistakes by adding a new feature to the TB detection classifiers. The initial X-ray images were used to extract the first set of features, and the edge-detected image was used to extract the second. Two publicly accessible datasets were utilized to assess the suggested method. The findings demonstrate that the suggested ensemble technique achieved the best accuracy, sensitivity, and specificity values of 89.77, 90.91, and 88.64%. This suggests that the detection rate for SEDL can be increased by utilizing various characteristics retrieved from various images.

While the solution presented by Hooda et al. (2019) combines elements from AlexNet, GoogleNet, and ResNet. The study's main contribution is creating an ensemble capable of performing TB classification by training these structures from scratch. On a combined dataset created using openly accessible standard datasets, the suggested approach is trained and evaluated. The ensemble outperforms most of the known approaches, with an accuracy of 88.24% and an area under the curve of 0.93.

The classification of shoulder images uses information from X-ray images from computer tomography and magnetic resonance imaging. Ensemble deep learning models are employed. The project aims to categorize the images with the help of artificial intelligence to determine their condition. The work uses information from musculoskeletal radiographs to identify shoulder fractures and analyse results using an ensemble and 26 deep-learning models. The overall accuracy of the twenty-eight classification was calculated using Cohen's kappa. The best result, 0.6942, was attained using an ensemble of ResNet34, DenseNet169, DenseNet201, and a sub-ensemble of various convolution networks (Uysal et al., 2021). Ensemble of deep learning models is the idea behind the SEDL model.

As tuberculosis (TB) is a serious health issue with a history of high mortality, early diagnosis is crucial for early disease control. As a result, an investigation was conducted in 2021 to detect TB in X-ray images of the chest using the Ensemble Learning method in conjunction with hybrid feature descriptors. Using convolutional neural networks and ensemble learning, the author suggested a novel method for TB diagnosis that mixes hand-crafted features with deep features. The dataset was collected from Montgomery and Shenzhen for the system's critical assessment. The operational characteristics curve from the Shenzhen and Montgomery models reaches 0.99 and 0.97, respectively, showing the distinction of the Ensemble machine learning approach over the other classifier as a single unit in classifications (Ayaz et al., 2021). The SEDL model achieved an accuracy of 98.8%, which is less than that of 0.99%, which the authors achieved; this could have been attributed to using a mixture of handcrafted and deep learning features. SEDL only used deep learning features.

Chest X-Rays are used in the ensemble learning for COVID-19 detection. The use of the Ensemble technique in the establishment of the X-ray classification to detect COVID-19's pulmonary manifestation is established. The approach uses a customized convolutional neural network and ImageNet pre-trained models, and the dataset comes from publically accessible databases. The best forecasts from the most accurate models are blended using various Ensemble techniques for a more accurate performance evaluation. The outcome demonstrates a significant improvement in COVID-19 detection on dataset sets, with an accuracy rate of 99.01% and an area under the curve of 0.9972. Prediction accuracy has significantly improved due to the combination of iterative, Ensemble, and modality basis knowledge transfer which we have to look at to increase the rate of accuracy of the SEDL (Rajaraman et al., 2020).

Here, are some published papers that use stacked ensemble deep learning:

“A multichannel EfficientNet deep learning-based stacking ensemble approach for lung disease detection using chest X-ray images” by Ravi et al. (2023). This paper proposes a multichannel deep-learning approach for lung disease detection using chest X-rays. The proposed method uses a stacked ensemble of three EfficientNet models, each trained on a different channel of the chest X-ray image. The ensemble model achieves an accuracy of 98% for the detection of pneumonia, 99% for the detection of tuberculosis, and 98% for the detection of COVID-19.

“Stacking Ensemble and ECA-EfficientNetV2 Convolutional Neural Networks on Classification of Multiple Chest Diseases Including COVID-19” by Huang and Liao (2022), Stacking-ensemble model, which combines six pre-trained models: EfficientNetV2-B0, EfficientNetV2-B1, EfficientNetV2-B2, EfficientNetV2-B3, EfficientNetV2-S, and EfficientNetV2-M. Based on ECA-Net and EfficientNetV2, the second model is a self-designed model called ECA-EfficientNetV2. On chest X-ray and CT images, each model underwent ten-fold cross-validation. A second dataset, the COVID-CT dataset, was used to assess the efficacy of the suggested Stacking-ensemble and ECA-EfficientNetV2 models. The proposed ECA-EfficientNetV2 model performs the best on both the chest X-ray dataset and the chest CT dataset, with the highest Accuracy of 99.21%, Precision of 99.23%, Recall of 99.25%, F1-score of 99.20%, and (area under the curve) AUC of 99.51%. The Stacking-ensemble and ECA-EfficientNetV2 models show no appreciable differences for any of the five criteria.

“CT Image Classification Based on Stacked Ensemble Of Convolutional Neural Networks” by Shomanov et al. (2022). First, looked at the model performance of the newest deep learning architectures, such as Inception, VGGNet, MobileNet, Xception, and ResNet50. Then, we chose seven cutting-edge models to apply to the various open CT datasets (SARS-CoV-2 CT-Scan, USCD CT, and COVID-X dataset). A set of medical image sets was used to fine-tune the model parameters after they were transferred from the other domain. The final convolutional layers were stacked using a fully connected neural network to identify the generalized feature space. InceptionV3, VGG16, VGG19, MobileNetV2, Xception, ResNet, and DenseNet201 had the highest peak accuracy among the single CNN models that were fine-tuned: 0.96, 0.94, and 0.94, respectively. The suggested ensemble model outperforms every other model, achieving the highest performance across all three open CT datasets with a peak accuracy of 0.99%.

Predictive models are less successful due to physicians' frequent failure to order tests or record results. This problem is addressed by a novel XGBoost method (Zhang et al., 2020), which imputes missing laboratory values using an unsupervised prefilling procedure and supervised machine learning. The results demonstrate that the novel model outperforms baseline and cutting-edge models on 13 frequently collected laboratory test variables, improving imputation by over 20% on average.

According to recent findings (Zivkovic et al., 2022), the classification accuracy of COVID-19 chest X-ray images can be improved using a CNN model with an XGBoost classifier. Performance is improved by tuning XGBoost hyperparameters with a hybrid AOA. With a classification accuracy of roughly 99.39% and weighted average precision, recall, and F1-score of 0.993889, 0.993887, and 0.993887, respectively, the suggested method performs better than existing cutting-edge methods.

The research (Ramaneswaran et al., 2021) proposes categorizing acute lymphoblastic leukemia (ALL) from microscopic white blood cell images using a hybrid Inception v3 XGBoost model. The model employs the XGBoost model as the classification head and Inception v3 as the image feature extractor. According to experiments, an XGBoost classification head performs better than a softmax classification head at classifying data. The proposed hybrid model achieves a weighted F1 score of 0.986.

The Italian Federation of General Practitioners dataset is used in this study (Romeo and Frontoni, 2022) to present a machine learning approach called Hierarchical Priority categorization eXtreme Gradient Boosting for the priority categorization of COVID-19 vaccination delivery. The suggested approach enhances the effectiveness of classification. It is integrated into a clinical decision support system, now assisting General Practitioners in allocating vaccine administration priorities to their assistants.

Stacking can aid in the reduction of overfitting, which happens when a model performs well on the training data but fails to generalize to new, untried data. Stacked ensembles can lessen overfitting and enhance generalization performance by mixing many models with distinct biases, which is the idea behind implementing the SEDL model (Akinbo and Daramola, 2021). Another issue that frequently arises in machine learning models is the bias-variance trade-off, partly addressed by stacked ensemble learning, the SEDL model. Stacked ensembles can balance underfitting and overfitting by merging models with various biases, improving classification performance (Aboneh et al., 2022).

Reasons that could have contributed to high accuracy, besides the advantages of combined power in ensemble techniques, include:

- We maintained very little correlation between the base classifiers utilized. This will ensure that those classifiers' faults are also diversified. The base classifiers are anticipated to work in tandem to get superior classification results. Only classifiers trained on similar features were combined in most studies analyzed. The base classifiers' correlation error is high as a result of this.
- We maintained the original image size, as most researchers lowered the original image's size during training to save on computing costs. Even with the most potent GPU hardware, training a highly complicated model requires significant processing power compared to the original image size.
- For training, hundreds of thousands of images from each class must be collected. In doing so, a classifier will be more precise. The amount of training data that is readily available, however, is frequently subpar due to the small number of datasets. Because of this, scientists are looking for several solutions to create a reliable classifier.

To improve on the Blending Methods: Blending methods, as mentioned in one of the research results (Chen et al., 2020), can improve the interpretability of stacked ensembles. Blending involves combining different ensemble learning models to allow for better interpretability. By summarizing the predictions of multiple models, blending methods can provide a clearer understanding of the underlying patterns and relationships in the data.

Feature Selection: The ensemble can be simplified by selecting a subset of the most informative features, and the resulting predictions can be easier to interpret. Feature selection techniques aim to identify the most relevant columns in a dataset.

Using Simpler Models: Simpler models can be employed instead of using complex models in the ensemble. This can make the predictions more interpretable and easier to explain. For example, using linear models or decision trees as base estimators in the ensemble can help overcome issues like high variance and low accuracy.

In general, the decision between complicated and interpretable models is based on the particular specifications of the issue. Simpler models can give better interpretability and ease of explanation, while complex models may offer higher predicted accuracy. To attain accuracy and interpretability in machine learning, striking the correct balance between simplicity and complexity is essential.

Interpretability refers to understanding how a neural network makes its predictions. Inception v3, VGG16, and ResNet34 are all convolutional neural network architectures used for image classification tasks. Here are some advantages of each network used in the SEDL in terms of interpretability:

Inception v3 Factorized convolutions: This helps to reduce computational efficiency as it reduces the number of parameters involved in a network, which makes it easier to understand the network's behavior. Progressive architecture: The architecture of an Inception v3 network is progressively built, step-by-step, which makes it easier to understand how the network is processing the input (Kurama, 2020).

VGG16 Simple architecture: VGG16 has a simple architecture with only 16 layers, making it easier to understand how the network processes the input (Rao et al., 2021).

ResNet34 Residual connections: ResNet34 uses residual connections, which allow the network to learn the residual mapping between layers. This makes it easier to understand how the network processes the input (Yang et al., 2021b).

Overall, all three networks used as weak learners have advantages in terms of interpretability. Inception v3 has a progressive architecture that makes it easy to understand how the network processes the input. In contrast, VGG16 has a simple architecture that makes it easy to understand the network's behavior. ResNet34 uses residual connections, making it easier to understand how the network processes the input.

Challenges in using stacked ensemble models for medical image analysis include:

1. The need for many base models: Stacked ensemble models require many base models to achieve their full potential. This can be a challenge, as it can be time-consuming and computationally expensive to train many deep-learning models.
2. The need for a diverse set of base models: The base models in a stacked ensemble should be diverse to improve the ensemble's performance. This can be a challenge, as finding a diverse set of deep learning models relevant to the task at hand can be difficult.

## 6. Conclusion

Ensemble techniques show great potential. Ensemble methods often improve detection accuracy. An ensemble of several features might provide better detection results. An ensemble of different deep-learning techniques could also be considered because ensembles perform better if the errors of the base classifiers have a low correlation. Ensemble learning has proven effective and functional in many problem domains and with significant applications in medical imaging. Deep ensemble learning builds several classifiers or a set of basic learners and merges their output to lessen overall variance. Compared to using a single classifier or base learner, the accuracy is greatly increased when combined with a group of classifiers or base learners. A potent deep learning approach known as deep ensemble learning has demonstrated clear benefits in numerous applications. The generalization capacity of an ensemble can be significantly higher than that of a single learner by utilizing numerous learners.

The ensemble models, which apply pooling functions on top of various deep convolutional neural network architectures, work by minimizing bias and variance to improve the accuracy of models. Modern medical image classification pipelines frequently combine unique architectures or models that have undergone varied training to maximize performance. Utilizing the prediction data from several methodologies leads to greater inference quality and bias or error reduction.

The fact that the user cannot understand the knowledge acquired by ensembles is a fundamental flaw in existing ensemble methods. The direction of increasing the comprehensibility of ensembles is significant yet poorly unexplored. Another significant problem is that, even though diversity is known to be crucial in ensembles, there are currently no satisfactory diversity measurements. In our upcoming work, we attempt to overcome these challenges to increase ensemble learning's ability to contribute to more applications.

## Data availability statement

The original contributions presented in the study are included in the article/supplementary material, further inquiries can be directed to the corresponding author.

## Author contributions

All authors listed have made a substantial, direct, and intellectual contribution to the work and approved it for publication.
